# Comparison of machine learning models for predicting stroke risk in hypertensive patients: Lasso regression model, random forest model, Boruta algorithm model, and Boruta algorithm combined with Lasso regression model

**DOI:** 10.1097/MD.0000000000042690

**Published:** 2025-05-30

**Authors:** Junzhang Huang, Wencai Liu

**Affiliations:** a Department of General Surgery, Lianjiang Traditional Chinese Medicine Hospital, Zhanjiang, Guangdong, China; b Department of Orthopedics, Shanghai Sixth People’s Hospital Affiliated to Shanghai Jiao Tong University School of Medicine, Shanghai, China.

**Keywords:** Boruta algorithm, Lasso regression, machine learning, random forest, stroke

## Abstract

The aim of this study was to compare the performance of 4 machine learning models—Lasso regression model, random forest model, Boruta algorithm model, and the Boruta algorithm combined with Lasso regression—in predicting stroke risk among hypertensive patients. The study evaluated the strengths and weaknesses of each model to provide a more clinically valuable prediction model for stroke risk. The study included 3472 hypertensive patients, of which 312 had experienced a stroke, and 3160 had not. Various health indicators were analyzed using Lasso regression, random forest, Boruta algorithm, and the Boruta algorithm combined with Lasso regression. Model performance was evaluated based on the area under the curve (AUC) of the receiver operating characteristic curve, the precision–recall curve, calibration curve, and decision curve analysis to assess classification ability, precision, calibration, and clinical benefit. The Lasso regression and Boruta algorithm models both have an AUC of 0.716, making them the best-performing models in terms of classification ability. The Boruta algorithm combined with Lasso regression model has an AUC of 0.705, slightly lower than the previous 2 models but still shows good predictive capability, with better interpretability due to feature selection. The random forest model has an AUC of 0.626, which is the lowest among the models, indicating weaker classification performance compared to the others. Among the 4 models, the Lasso regression model and Boruta algorithm model performed similarly in terms of classification ability, both demonstrating moderate predictive power, while the random forest model performed relatively poorly. The Boruta combined with Lasso regression model was precise in variable selection but had limited clinical utility. Therefore, the Lasso regression model appears to be the most balanced in predicting stroke risk and is the recommended model based on this study.

## 1. Introduction

Hypertension is one of the most common chronic diseases globally and a major risk factor for stroke.^[1]^ The occurrence of stroke is not only related to hypertension, but also affected by age, gender, body mass index, diabetes, cardiovascular diseases, respiratory diseases, and other factors. In recent years, with the development of machine learning technology, more and more studies have begun to attempt to use these methods to establish more accurate disease prediction models.^[2–5]^

Different machine learning models have their own advantages in dealing with multivariate and nonlinear relationships, so choosing the appropriate model is crucial for stroke risk prediction.^[6,7]^ However, there is still room for improvement in accuracy and calibration of these models at present. Future research should further introduce more clinical factors and optimize the calibration methods of the model to improve predictive performance.

This study compared the performance of 4 common machine learning models, namely Lasso regression, random forest, Boruta algorithm, and Boruta combined with Lasso regression, in predicting stroke risk in hypertensive patients. We evaluate the accuracy of classification, model stability, and clinical application value, analyze the advantages and disadvantages of each model, and aim to provide reference for more accurate identification of high-risk patients and personalized intervention strategies in clinical practice,^[8–10]^ helping to reduce stroke incidence and improve patient outcomes.^[11–13]^

## 2. Methods

### 2.1. Data source

The data used in this study comes from National Health and Nutrition Examination Survey, a large-scale health and nutrition monitoring program led by the National Center for Health Statistics and aimed at residents nationwide, covering a wide range of demographic characteristics, health status, and lifestyle information. The data analyzed in this study was taken from 2 survey cycles from 2005 to 2008. Patients were screened based on inclusion and exclusion criteria, and a total of 3472 individuals diagnosed with hypertension were included. Inclusion criteria: Participants diagnosed with hypertension based on self-report or clinical blood pressure readings; Complete stroke history assessment through standardized questionnaires; Provide complete data on important variables related to stroke risk prediction. Exclusion criteria: individuals without diagnosed hypertension; Individuals with missing or incomplete key variables (such as history of stroke or hypertension); According to clinical reviews, individuals with extreme outliers are considered unreliable.

All research plans have been approved by the Ethics Review Committee of the National Center for Health Statistics, and the relevant procedures follow local laws, regulations, and institutional requirements. All participants signed informed consent forms before the start of the study to ensure ethical compliance.

### 2.2. Variables

Include these variables for analysis: age, height, weight, body mass index, gender, diabetes, asthma, overweight, arthritis, heart failure, coronary heart disease, angina pectoris, heart attack, emphysema, chronic bronchitis, liver condition, cancer, and stroke.

### 2.3. Statistical tools

Before conducting statistical analysis, we first systematically cleaned and preprocessed the data to ensure data quality and the reliability of the analysis results. Adopt appropriate imputation methods to fill in missing values in variables to reduce the impact of potential bias. After conducting normality tests on continuous variables, if they follow a normal distribution, they are expressed as mean ± standard deviation; If it does not follow a normal distribution, describe it as median and interquartile range. The categorical variables are presented in the form of frequency and percentage. In inter group comparisons, appropriate statistical methods are selected based on the type of variable and its distribution characteristics: for continuous variables that follow a normal distribution and have homogeneity of variance, independent sample *t* tests are used to compare the differences in mean values between the 2 groups; If normality or homogeneity of variance is not satisfied, nonparametric testing should be used instead. The comparison between categorical variables is conducted using chi square test. All tests were conducted using a two-sided test, with a *P* value < .05 considered statistically significant.

In addition, we conducted further advanced statistical modeling and multi factor analysis using R language (version 4.4.1) and its online analysis platform, including data preprocessing, variable screening, model construction, and performance evaluation.

### 2.4. Machine learning models

This study used 4 models to evaluate the predictive ability of stroke risk: Lasso regression achieves feature selection by penalizing coefficients to prevent overfitting; Random forest utilizes multiple decision trees to handle nonlinear relationships and ranks variables based on their importance; The Boruta algorithm identifies all relevant variables by comparing them with random features and is suitable for high-dimensional data; The Boruta algorithm combined with Lasso regression model first screens important features, and then performs regularization, combining the advantages of both to improve model performance. To comprehensively evaluate model performance, several metrics were applied.

The area under the curve (AUC) of the receiver operating characteristic (ROC) curve assessed the model’s ability to distinguish between stroke and non-stroke patients, with values closer to 1 indicating better classification. The precision–recall (PR) curve evaluated the balance between precision and recall, particularly valuable in the context of imbalanced datasets. The calibration curve measured the agreement between predicted probabilities and actual outcomes, where ideal calibration aligns with the 45-degree diagonal. Lastly, decision curve analysis (DCA) was used to assess the clinical utility of each model by examining the net benefit across various probability thresholds.

## 3. Result

### 3.1. Patient baseline data

This study included a total of 3472 hypertensive patients, among whom 312 hypertensive patients experienced stroke. There were significant differences in age, height, weight, diabetes, arthritis, heart failure, coronary heart disease, angina pectoris, heart attack, emphysema, chronic bronchitis, and cancer between the stroke group and the non-stroke group in hypertension patients (*P* < .05) (Table 1).

**Table 1:** Patient demographics and baseline characteristics.

Characteristic	Stroke		*P*-value
	No, N = 3160*	Yes, N = 312*	
Age (yr)	60 ± 15	68 ± 12	<.001†
Height (cm)	167 ± 10	165 ± 9	.005†
Weight (kg)	86 ± 23	83 ± 18	.003†
BMI (kg/m^2^)	31 ± 7	30 ± 6	.113†
Gender			.852‡
Female	1674 (53.0%)	167 (53.5%)	
Male	1486 (47.0%)	145 (46.5%)	
Diabetes			<.001‡
No	2455 (77.7%)	192 (61.5%)	
Yes	705 (22.3%)	120 (38.5%)	
Asthma			.242‡
No	2663 (84.3%)	255 (81.7%)	
Yes	497 (15.7%)	57 (18.3%)	
Overweight			.690‡
No	1654 (52.3%)	167 (53.5%)	
Yes	1506 (47.7%)	145 (46.5%)	
Arthritis			<.001‡
No	1776 (56.2%)	120 (38.5%)	
Yes	1384 (43.8%)	192 (61.5%)	
Heart failure			<.00‡
No	2955 (93.5%)	252 (80.8%)	
Yes	205 (6.5%)	60 (19.2%)	
Coronary heart disease			<.001‡
No	2922 (92.5%)	255 (81.7%)	
Yes	238 (7.5%)	57 (18.3%)	
Angina pectoris			<.001‡
No	2991 (94.7%)	270 (86.5%)	
Yes	169 (5.3%)	42 (13.5%)	
Heart attack			<.001‡
No	2931 (92.8%)	242 (77.6%)	
Yes	229 (7.2%)	70 (22.4%)	
Emphysema			.003‡
No	3059 (96.8%)	292 (93.6%)	
Yes	101 (3.2%)	20 (6.4%)	
Chronic bronchitis			.004‡
No	2904 (91.9%)	272 (87.2%)	
Yes	256 (8.1%)	40 (12.8%)	
Liver condition			.941‡
No	3011 (95.3%)	297 (95.2%)	
Yes	149 (4.7%)	15 (4.8%)	
Cancer			<.001‡
No	2719 (86.0%)	244 (78.2%)	
Yes	441 (14.0%)	68 (21.8%)	

* Mean ± SD; n (%).

† Welch two sample *t* test.

‡ Pearson chi-squared test.

### 3.2. Lasso regression model

**Figure 1.**
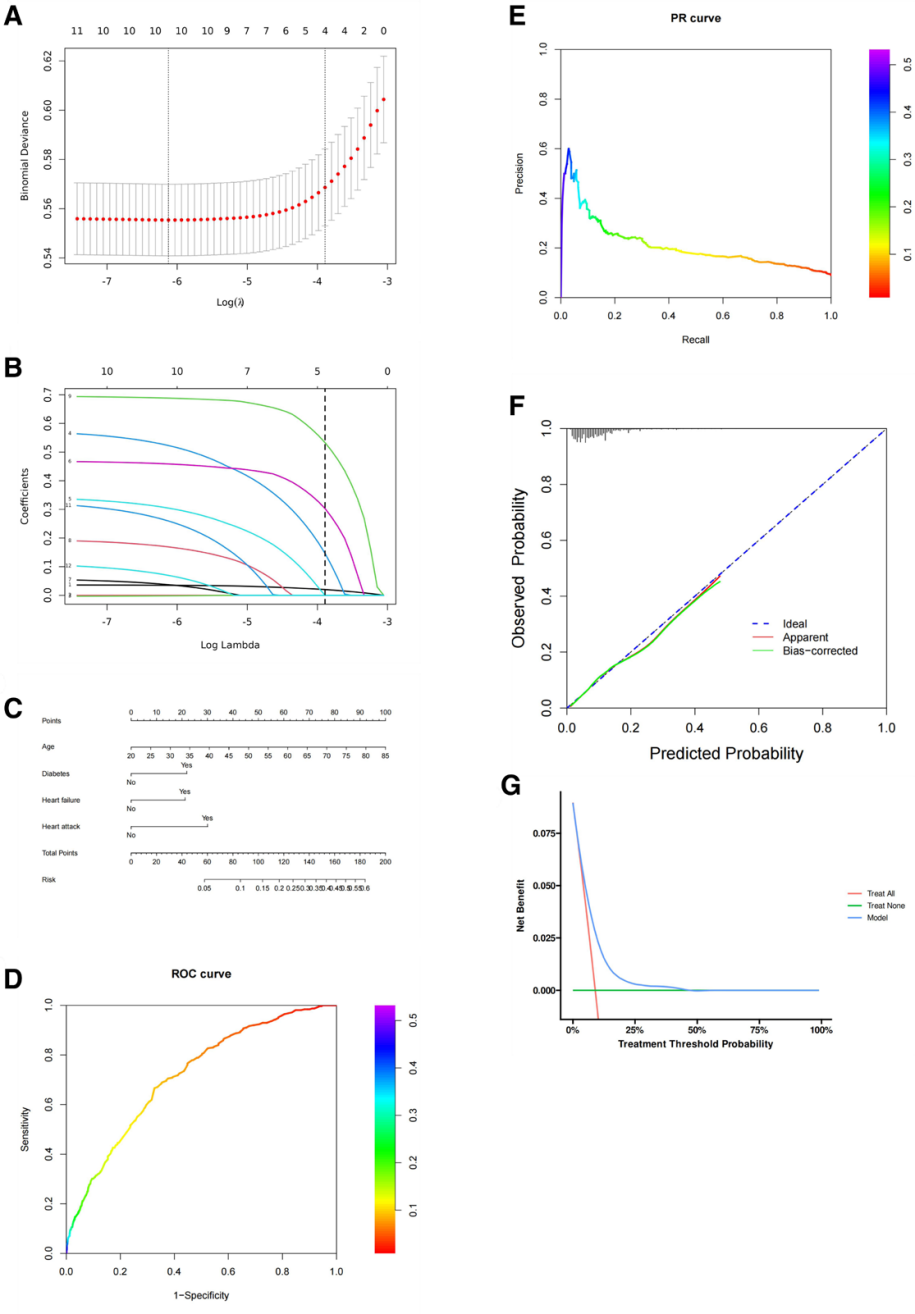
Lasso regression model. (A) Lasso regression cross-validation plot. (B) Lasso regression variable selection path plot. (C) Nomogram. (D) ROC curve. (E) PR curve. (F) Calibration curve. (G) DCA curve.

Figure 1A is used to display the model performance of Lasso regression at different levels of regularization, such as binomial deviance. Through cross-validation, the optimal λ value can be identified, ensuring the model performs well on both training and validation datasets. Figure 1B shows how the coefficients of variables change as the regularization parameter λ increases. Some coefficients may shrink to zero, indicating that these variables are excluded from the model under stricter regularization, which is helpful for variable selection. Table 2 displays variables selected by Lasso regression and then analyzed through logistic regression. Age, diabetes, heart failure, and heart attack have odds ratios and 95% confidence intervals listed, with *P*-values all <.05, indicating a significant association with the dependent variable.

**Table 2:** Lasso regression with variable reduction.

Characteristic	N	Event N	OR	95% CI	*P*-value
Age	3472	312	1.04	1.03, 1.05	<.001
Diabetes					
No	2647	192	–	–	
Yes	825	120	1.79	1.39, 2.30	<.001
Heart failure					
No	3207	252	–	–	
Yes	265	60	1.75	1.23, 2.51	.002
Heart attack					
No	3173	242	–	–	
Yes	299	70	2.22	1.59, 3.10	<.001

CI = confidence interval, OR = odds ratio.

In Figure 1C, the nomogram typically illustrates the relationship between the model’s predicted probabilities and the actual occurrence of events. The chart may include risk scores for different variable combinations and their corresponding predicted probabilities. The ROC curve is used to assess the model’s classification ability, with an AUC of 0.716, indicating moderate classification performance (Fig. 1D). The PR curve evaluates the trade-off between precision and recall at different thresholds, with an AUC of 0.209, suggesting that the model’s precision and recall may not be ideal at certain thresholds (Fig. 1E). The calibration curve assesses the consistency between the model’s predicted probabilities and the actual occurrence probabilities (Fig. 1F). Ideally, the calibration curve should approach the diagonal line, indicating that the predicted probabilities align with the actual probabilities. DCA evaluates the net benefit of the model at different treatment thresholds (Fig. 1G). The curve can assist decision-makers in selecting the optimal treatment threshold to maximize patient health benefits.

### 3.3. Random forest model

**Figure 2.**
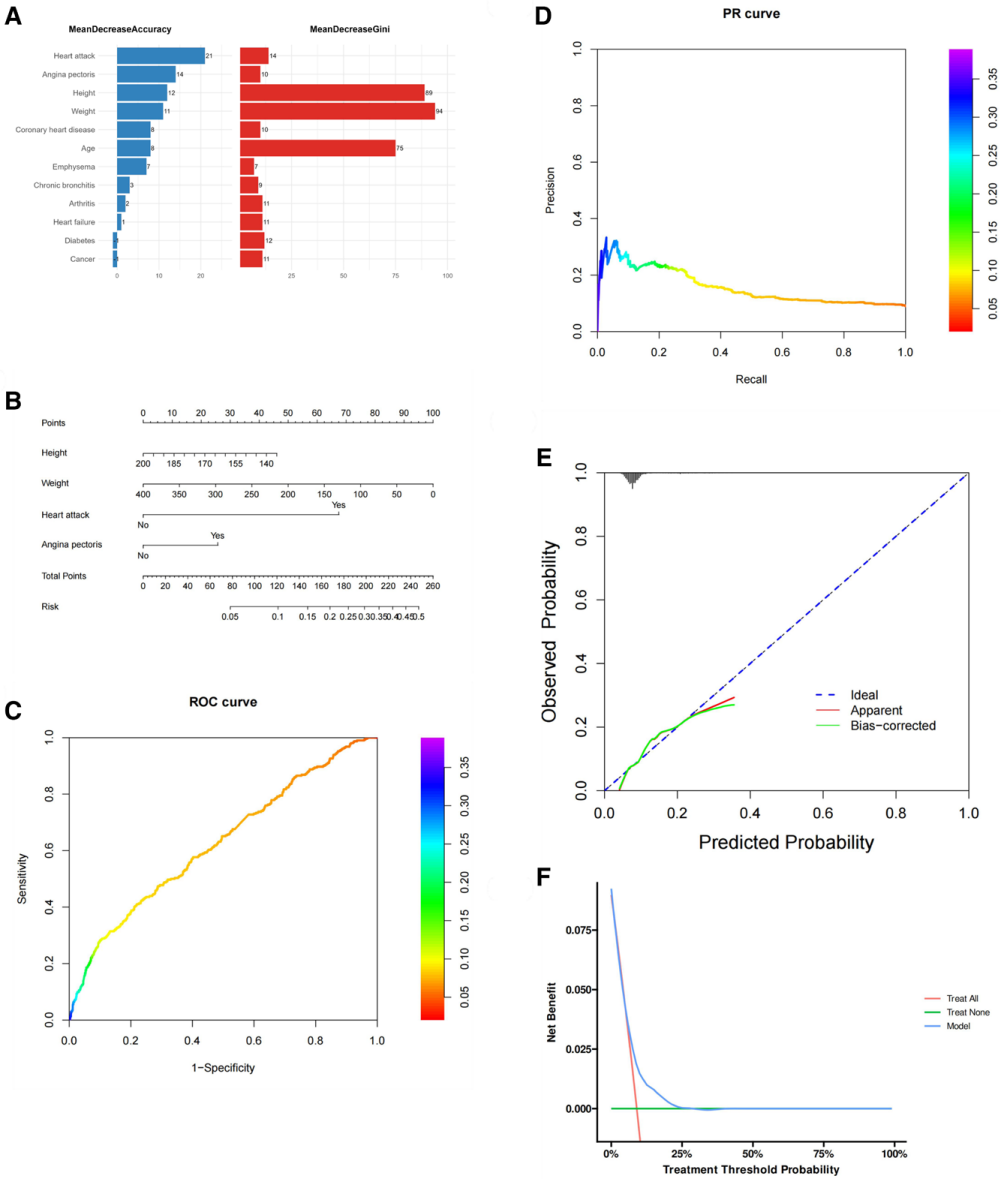
Random forest model. (A) Variable sorting chart. (B) Nomogram. (C) ROC curve. (D) PR curve. (E) Calibration curve. (F) DCA curve.

Figure 2A shows the importance of variables in terms of “Mean Decrease Accuracy” and “Mean Decrease Gini.” These values help us rank the variables based on their contribution to the model’s performance (Table 3). The selected variables are used to build a nomogram, with each variable having a corresponding scale to help us estimate a person’s total risk score, and further predict the risk of stroke in hypertensive patients (Fig. 2B). The AUC of the ROC curve is 0.626, indicating that the model’s ability to distinguish between categories is moderate. The closer the AUC value is to 1, the better the model’s performance, while a value closer to 0.5 suggests poor model performance (Fig. 2C). The PR curve shows the relationship between precision and recall (Fig. 2D). The calibration curve compares predicted probabilities with observed probabilities (Fig. 2E). The ideal line represents perfect calibration, where predicted probabilities match actual outcomes. The model appears to underpredict at lower probabilities but slightly improves at higher probabilities, indicating some level of bias in its predictions. The DCA curve evaluates the net benefit of using the model for decision-making across different thresholds of probability (Fig. 2F).

**Table 3:** Variable value ranking table.

Variables	Mean decrease accuracy	Mean decrease gini
Heart attack	20.6623143	13.784896
Angina pectoris	14.0377725	9.821881
Height	12.3923678	89.019640
Weight	11.1598157	94.195858
Coronary heart disease	8.2905105	9.517542
Age	8.0485720	75.185164
Emphysema	7.1667282	7.151413
Chronic bronchitis	2.6704075	9.471501
Arthritis	1.7315612	11.455196
Heart failure	0.5881645	10.896726
Diabetes	‐0.6704060	11.600159
Cancer	‐1.1077690	10.983067

### 3.4. Boruta algorithmic model

**Figure 3.**
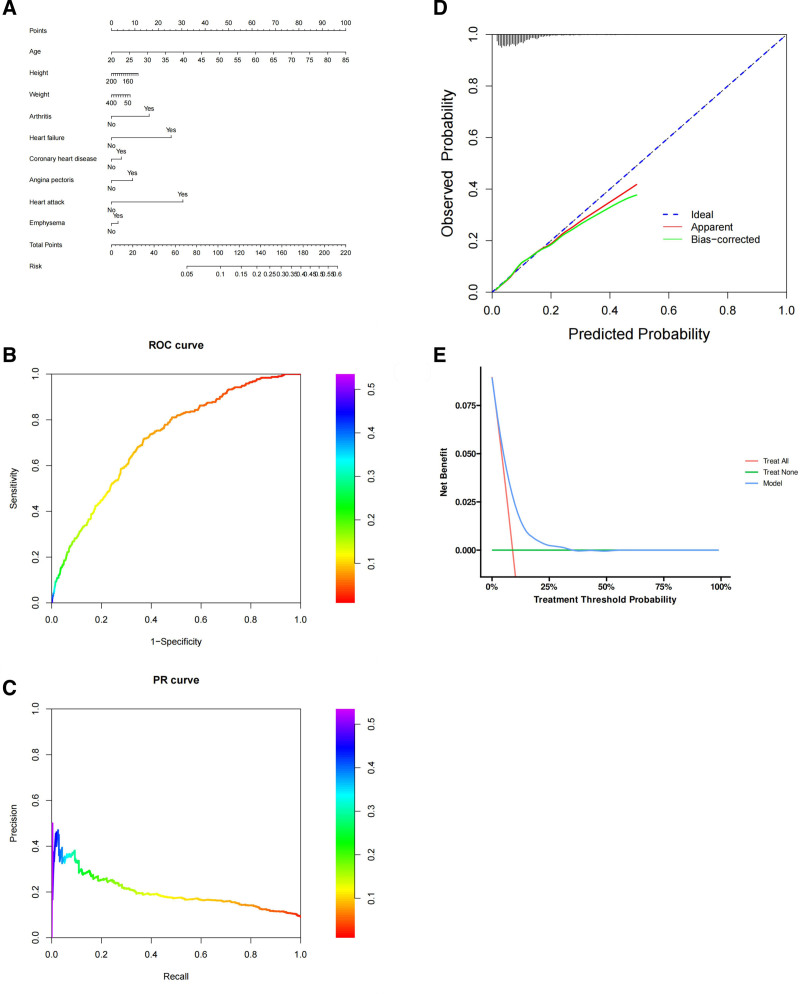
Boruta algorithmic model. (A) Nomogram. (B) ROC curve. (C) PR curve. (D) Calibration curve. (E) DCA curve.

Variables such as heart attack, angina pectoris, coronary heart disease, age, height, weight, heart failure, emphysema, and arthritis have been confirmed as important, meaning they play significant roles in the model (Table 4). The risk value can be estimated using the scale at the bottom of the chart (Fig. 3A). The ROC curve assesses the model’s classification performance (Fig. 3B). The vertical axis represents sensitivity, while the horizontal axis shows 1-specificity. The AUC is 0.716, indicating moderate classification ability. The PR curve evaluates model performance in the context of imbalanced data (Fig. 3C). In this chart, the model’s predictions slightly overestimate the event likelihood, especially in the mid-to-high probability range, indicating a need for further calibration of the predicted probabilities (Fig. 3D). The chart shows that the model offers some net benefit at lower thresholds, but the net benefit declines rapidly at higher thresholds, indicating limited utility at higher decision thresholds (Fig. 3E).

**Table 4:** Variable selection with Boruta algorithm.

Variables	Mean importance	Median importance	Min importance	Max importance	Norm hits	Final decision
Heart attack	19.6688228	19.45161149	16.145959	23.166987	1.00000000	Confirmed
Angina pectoris	14.0764799	14.04062994	11.039642	17.440826	1.00000000	Confirmed
Coronary heart disease	11.1719598	11.16181905	8.198546	14.329826	1.00000000	Confirmed
Age	10.7360890	10.64050434	7.143172	14.413265	1.00000000	Confirmed
Height	10.2098021	10.15927479	7.137833	13.378053	1.00000000	Confirmed
Weight	9.8215122	9.69244625	6.707964	13.470636	1.00000000	Confirmed
Heart failure	6.7060419	6.46586111	3.365577	11.749744	0.96969697	Confirmed
Emphysema	6.1219642	6.32953471	1.365717	8.719113	0.96969697	Confirmed
Arthritis	5.6327872	5.48369348	2.836808	9.688156	0.97979798	Confirmed
Chronic bronchitis	2.0759203	2.10692727	‐2.056943	5.627270	0.43434343	Tentative
Diabetes	2.0641191	2.04860923	‐2.120069	5.269905	0.41414141	Tentative
Cancer	0.3051721	0.06199269	‐1.538694	2.732555	0.01010101	Rejected

### 3.5. Boruta algorithm + Lasso regression

**Figure 4.**
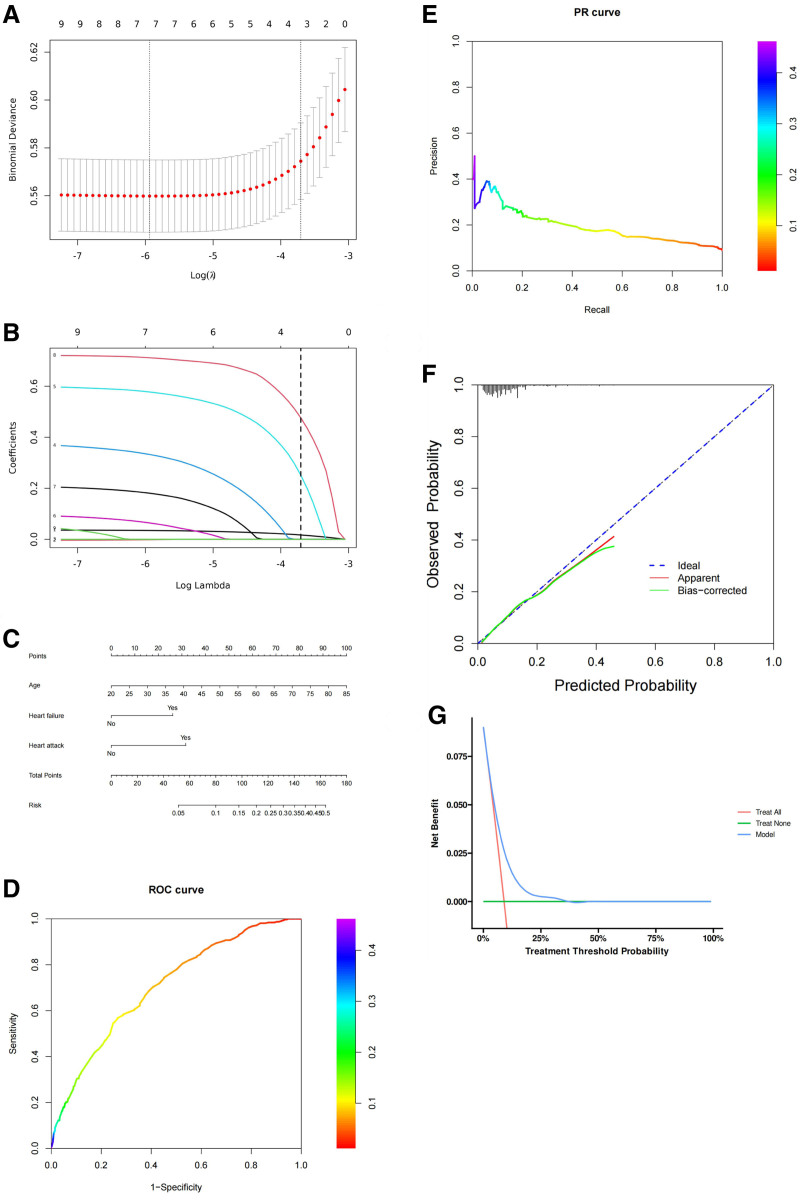
Boruta algorithm + Lasso regression. (A) Lasso regression cross-validation plot. (B) Lasso regression variable selection path plot. (C) Nomogram. (D) ROC curve. (E) PR curve. (F) Calibration curve. (G) DCA curve.

The variables selected using the Boruta algorithm were further included in Lasso regression analysis for additional variable selection (Table 4). Figure 4A is a cross-validation plot of Lasso regression, showing the average error of the model at different λ values, which helps to select the optimal λ value. Figure 4B is the variable selection path diagram of Lasso regression, showing the changes in the coefficients of each variable with the variation of λ value, and indicating which variables are gradually removed or retained. The nomogram visually represents the final model based on the selected variables after Lasso regression (Fig. 4C). The AUC value of the ROC curve in the chart is 0.705 (Fig. 4D). The PR curve evaluates the model’s performance, especially on imbalanced datasets (Fig. 4E). The calibration curve assesses how well the predicted probabilities align with actual outcomes (Fig. 4F). Ideally, the curve should follow the diagonal blue dashed line, representing perfect calibration. In this case, the green and red lines deviate slightly, indicating that the model may overestimate or underestimate risk in some ranges. This suggests room for improvement in predicting accurate probabilities. At lower thresholds, the model offers some net benefit, but this benefit diminishes as the threshold increases, indicating the model’s limited clinical utility at higher decision thresholds (Fig. 4G).

### 3.6. Model comparison

**Figure 5.**
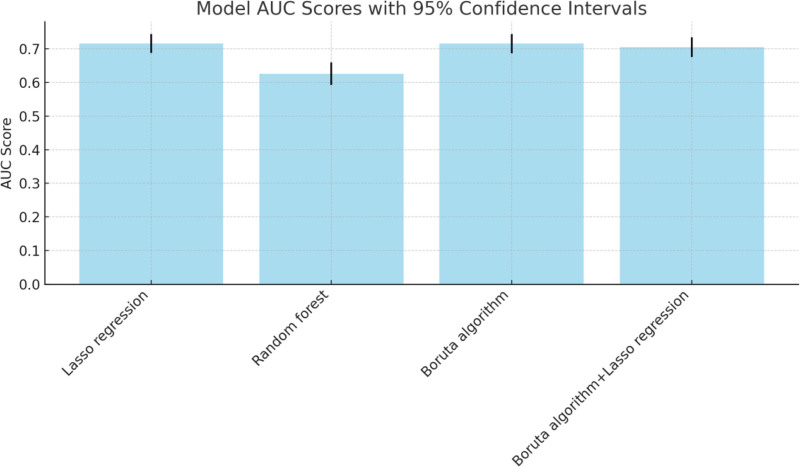
Model comparison.

The Lasso regression and Boruta algorithm models both have an AUC of 0.716, making them the best-performing models in terms of classification ability. They are well-suited for complex datasets (Table 5 and Fig. 5). The Boruta algorithm combined with Lasso regression model has an AUC of 0.705, slightly lower than the previous 2 models but still shows good predictive capability, with better interpretability due to feature selection. The random forest model has an AUC of 0.626, which is the lowest among the models, indicating weaker classification performance compared to the others.

**Table 5:** Model comparison table.

Model	AUC	95% CI
Lasso regression	0.716	0.688–0.744
Random forest	0.626	0.592–0.660
Boruta algorithm	0.716	0.687–0.744
Boruta algorithm + Lasso regression	0.705	0.676–0.734

AUC = area under the curve, CI = confidence interval.

## 4. Discussion

This study analyzed 3472 hypertensive patients, of whom 312 had suffered a stroke. We used 4 machine learning methods: Lasso regression, random forest, Boruta algorithm, and a model that combines Boruta and Lasso regression. Through these models, we evaluated their ability to predict stroke risk in hypertensive patients, with the aim of providing more accurate predictive tools for clinical practice.

We considered the impact of sample size on model training and validation in our research to ensure the reliability of the results. The minimum sample size of a model is influenced by multiple factors, including the complexity of the model, the number of predictive factors, and the required statistical power. Usually, larger sample sizes can improve the stability and applicability of models, especially for complex models such as random forests and Boruta. In this study, the sample size of 3472 hypertensive patients was sufficient to ensure the reliability of the results, although increasing the sample size may further improve the performance of the model and reduce potential bias.

The performance of these machine learning and statistical models is affected by some assumptions and limitations. For example, Lasso regression performs well in feature selection, but when variables are highly correlated, it may compress some valuable predictor coefficients to zero, thereby ignoring the interactions between variables. Random forests may be very sensitive to the size of the training set, and overfitting or underfitting may occur when dealing with small or imbalanced data, affecting the performance of the model. Although the Boruta algorithm is useful in feature selection, it may not be as effective in capturing the interactions between variables as other methods, especially when combined with Lasso regression, where the model may lose some flexibility. These characteristics require us to pay special attention to the applicability and limitations of the model when selecting suitable stroke risk prediction methods.

The poor performance of the random forest model (AUC = 0.626) may be related to several factors. Firstly, when facing imbalanced or high-dimensional data, the model may experience overfitting, especially without effective regularization. Secondly, improper hyperparameter settings, such as the number of trees, maximum depth, or minimum sample size per leaf, may also affect the performance of the model. In addition, the high correlation between features may reduce the discriminative ability of the model. To improve model performance, in the future, hyperparameters can be optimized through grid search to reduce the correlation between features, and cross-validation can be used to enhance the stability and generalization ability of the model.

The Boruta algorithm mainly identifies variables that make significant contributions to the target variable by iterating over them and comparing them. The AUC of the ROC curve is 0.716, indicating moderate classification performance. In an imbalanced dataset, the PR curve evaluates the balance between accuracy and recall. In this model, the PR curve indicates that the model may slightly overestimate the likelihood of events in the medium to high probability range, suggesting that there is room for optimization balance between accuracy and recall. The calibration curve shows that the model may underestimate risk when predicting low probability events, although it performs slightly better at higher probabilities. Overall, the calibration of the model is not perfect, indicating that in some cases, it may overestimate or underestimate risk. The DCA curve indicates that the model provides some net benefits at lower thresholds, but as the threshold increases, the benefits rapidly decrease, indicating limited clinical applicability of the model at higher decision thresholds. The Boruta algorithm model performs well in the initial variable selection process and can identify features that make significant contributions to predicting the target variable.^[14,15]^ However, although the AUC of the ROC curve is 0.716, the PR curve and calibration curve indicate that the model is difficult to handle imbalanced data. To improve performance, further optimizing variable selection or introducing other regularization techniques can enhance the predictive accuracy and stability of the model.

Although the combination of Boruta and Lasso regression performed well in predictive performance (AUC = 0.705), its performance slightly decreased compared to using Lasso regression or Boruta models alone (both AUC = 0.716). This difference may be caused by several reasons. Firstly, although Boruta can effectively identify relevant features, it may also retain some weakly correlated variables. On the other hand, Lasso regression automatically selects variables and shrinks them,^[16–18]^ and combining them with Boruta may lead to excessive constraints on the feature space, thereby ignoring the small but useful interactions that may exist between predictor variables. In addition, integrated models may introduce redundancy and even weaken the synergistic effects between different features, affecting overall performance. Therefore, although the Boruta algorithm combined with the Lasso regression model improves the interpretability of feature selection, it may also have a slight impact on predictive ability.

These machine learning models can assist clinicians in conducting quantitative stroke risk assessments for hypertensive patients, thereby supporting clinical decision-making. Lasso regression and Boruta algorithm showed moderate predictive ability (AUC = 0.716) and are easy to integrate into clinical practice, helping to identify high-risk patients who may benefit from early intervention. By using these models, doctors can better stratify patient risk, prioritize resource allocation for high-risk patients, optimize treatment plans, such as adjusting antihypertensive drugs or increasing monitoring frequency.

Although the combination of Boruta and Lasso regression provides better feature selection and interpretability, due to its complexity, it may not be as direct as a single Lasso regression model in clinical applications. Although the random forest model has low predictive ability, it may still be suitable for certain clinical environments with large amounts of data and low interpretability requirements.^[19–21]^ Overall, combining these models and integrating them into routine clinical procedures can help doctors concentrate resources and provide personalized treatment plans for patients at high risk of stroke, thereby improving patient prognosis and reducing medical costs.

## 5. Conclusion

Overall, Lasso regression and Boruta algorithm excel in AUC and model simplification, making them ideal for tasks requiring high accuracy and feature selection. The Boruta combined with Lasso regression model offers a balanced approach, maintaining good performance while improving interpretability through enhanced feature selection. In contrast, the random forest model underperforms in this dataset and, although generally robust, is not the best choice in this scenario.

## Author contributions

**Conceptualization:** Junzhang Huang.

**Data curation:** Junzhang Huang.

**Formal analysis:** Junzhang Huang.

**Investigation:** Junzhang Huang.

**Supervision:** Wencai Liu.

**Validation:** Wencai Liu.

**Visualization:** Wencai Liu.
